# Feasibility of deescalating postoperative care in enhanced recovery after cardiac surgery

**DOI:** 10.3389/fcvm.2024.1412869

**Published:** 2024-08-12

**Authors:** Sina Stock, Sarah Berger Veith, Theresa Holst, Sahab Erfani, Julia Pochert, Christian Dumps, Evaldas Girdauskas

**Affiliations:** ^1^ Department of Cardiac and Thoracic Surgery, University Hospital Augsburg, Augsburg, Germany; ^2^ Department of Anesthesiology and Intensive Care Medicine, University Hospital Augsburg, Augsburg, Germany

**Keywords:** enhanced recovery after surgery (ERAS), enhanced recovery after cardiac surgery (ERACS), minimally invasive cardiac surgery (MICS), heart valve surgery, postoperative care, post-anesthesia care unit (PACU), on-table extubation

## Abstract

**Introduction:**

Enhanced Recovery After Surgery (ERAS) prioritizes faster functional recovery after major surgery. An important aspect of postoperative ERAS is decreasing morbidity and immobility, which can result from prolonged critical care. Using current clinical data, our aim was to analyze whether a six-hour monitoring period after Minimally Invasive Cardiac Surgery (MICS) might be sufficient to recognize major postoperative complications in a future Fast Track pathway. Additionally, we sought to investigate whether it could be possible to deescalate the setting of postoperative monitoring.

**Methods:**

358 patients received MICS and were deemed suitable for an ERAS protocol between 01/2021 and 03/2023 at our institution. Of these, 297 patients could be successfully extubated on-table, were transferred to IMC or ICU in stable condition and therefore served as study cohort. Outcomes of interest were incidence and timing of Major Adverse Cardiac Events (MACE; death, myocardial infarction requiring revascularization, stroke), bleeding requiring reexploration and Fast Track-associated complications (reintubation and readmission to ICU).

**Results:**

Patients' median age was 63 years (IQR 55–70) and 65% were male. 189 (64%) patients received anterolateral mini-thoracotomy, primarily for mitral and/or tricuspid valve surgery (*n* = 177). 108 (36%) patients had partial upper sternotomy, primarily for aortic valve repair/replacement (*n* = 79) and aortic surgery (*n* = 17). 90% of patients were normotensive without need for vasopressors within 6 h postoperatively, 82% of patients were transferred to the general ward on postoperative day 1 (POD). Two (0.7%) MACE events occurred, as well as 4 (1.3%) postoperative bleeding events requiring reexploration. Of these complications, only one event occurred before transfer to the ward - all others took place on or after POD 1. There was one instance of reintubation and two of readmission to ICU.

**Conclusions:**

If MICS patients can be successfully extubated on-table and are hemodynamically stable, major postoperative complications were rare in our single-center experience and primarily occurred after transfer to the ward. Therefore, in well selected MICS patients with uncomplicated intraoperative course, monitoring for six hours, possibly outside of an ICU, followed by transfer to the ward appears to be a feasible theoretical concept without negative impact on patient safety.

## 1 Introduction

In recent years, cardiac surgery has undergone a paradigm shift towards minimally invasive techniques, aiming to reduce surgical trauma, enhance patient recovery and optimize healthcare resources. Alongside surgical advancements, perioperative care strategies have evolved, with enhanced recovery after surgery (ERAS) protocols emerging as a cornerstone for improving postoperative outcomes.

ERAS represents a comprehensive, interprofessional protocol aimed at optimizing patient care following major surgery. Its primary objectives include reducing surgical stress, minimizing complications and accelerating postoperative recovery, all with the ultimate goal of swiftly restoring patients to their preoperative functional baseline. ERAS, originating in colorectal surgery in the 1990s, has expanded across different surgical specialties. Since the early 2000s, ERAS principles have also been tailored for the field of cardiac surgery, incorporating the complexities of cardiac surgical procedures and their potential for postoperative morbidity. The establishment of the ERAS Cardiac Society in 2017 marked a significant milestone, providing evidence-based guidelines for ERAS programs in cardiac surgery (1). The interventions detailed in the guideline have led to reduced complications, shorter hospital stays, decreased healthcare costs and improved patient satisfaction, all while maintaining safety standards (2–5).

One key element of ERAS in cardiac surgery is the optimization of postoperative monitoring and management, particularly regarding the intensity and duration of care provided in the immediate postoperative period. Traditionally, all patients undergoing cardiac surgery have been routinely admitted to the intensive care unit (ICU) for overnight observation, despite the absence of immediate postoperative complications. In recent decades, however, the increasing shortage of available ICU beds has led to the introduction of dedicated cardiac surgery recovery units, referred to as “intermediate cardiothoracic surgical wards (IMC)” (6), “Fast Track units” (7) or “post-anesthesia care units (PACU)” (8). These units provide invasive monitoring, but reduced staff to patient ratio, thereby saving ICU capacity. Evaluation of these so-called Fast Track concepts has demonstrated adequate patient safety with low rates of readmission to ICU (9–12). Haanschoten et al. even further challenged the concept of Fast Track recovery in cardiac surgery by transferring patients to the general ward on the same day of the operation after a mean postoperative surveillance period of 7 h in a PACU (13). Based on the rapid evolution of minimally invasive cardiac surgery (MICS) procedures in reducing surgical trauma, blood loss and pain (14), the question arises whether a shorter, yet comprehensive, monitoring period in a PACU might nowadays indeed suffice to detect and manage postoperative complications effectively. This would not only align with the principles of ERAS but also offer potential further benefits in terms of more optimized resource utilization, patient comfort and healthcare cost reduction.

Based on the data collected from our ERAS program for MICS, in which patients still remained on the ICU or IMC overnight, we sought to systematically investigate whether, in the future, a six-hour monitoring period in a dedicated postoperative recovery unit (i.e., PACU) following MICS procedures could adequately identify major postoperative complications—thereby possibly obviating the need for an overnight IMC/ICU stay without compromising patient safety. By evaluating the feasibility and safety of this postoperative care pathway, we aim to contribute insights towards optimizing perioperative management in cardiac surgery, fostering patient-centered care and enhancing healthcare resource utilization.

## 2 Methods

### 2.1 Study population

We conducted a retrospective observational study of adult patients (>18 years of age) receiving MICS and deemed suitable for our institutional ERAS program. The study was conducted in accordance with the Declaration of Helsinki (2008) and ethics approval was received from the ethics committee of Ludwig-Maximilians-University Munich, Germany (Number 23-0915). All patients who received MICS between 01/2021 and 03/2023 and who had been marked preoperatively for our ERAS scheme were included in a retrospective database. Exclusion criteria for ERAS were emergency procedures, redo operations, those patients unwilling to participate in ERAS and those patients unable to achieve compliance with ERAS interventions due to neurological or physical limitations (e.g., known alcohol use disorder, cognitive impairment, inability to walk preoperatively). Due to the retrospective nature of this study, no written consent was required. The selected patients underwent standardized data collection for demographic, intra- and postoperative data.

For the purpose of this study, only patients who could be extubated on-table and who were hemodynamically stable were considered. Patients with significant hemodynamic instability (e.g., moderate or higher dose vasopressors), respiratory instability or complicated intraoperative course (e.g., bleeding, technical complications) and—as a consequence thereof—medical indication for ICU admission were excluded from this analysis, even if successful on-table extubation could be achieved.

### 2.2 Study design

Outcomes of interest were the incidence and timing of Major Adverse Cardiac Events (MACE; death, myocardial infarction requiring revascularization, stroke), bleeding requiring reexploration, need for vasoactive drugs and potentially Fast Track-associated complications (i.e., reintubation and readmission to ICU). The goal of this analysis was to better understand the immediate postoperative time window as it pertains to complications including bleeding, vasoplegia and respiratory insufficiency. We further investigated whether monitoring patients for >6 h in a high-care unit significantly increases the safety profile and whether it would therefore be feasible to transfer them to the ward earlier than the currently implemented overnight stay.

### 2.3 Patient flow in the ERAS program

Our institution's ERAS program is implemented for most patients undergoing MICS, with patient selection based on individual risk assessment by a senior surgeon. The ERAS approach starts with prehabilitation, which is achieved through a preoperative interdisciplinary clinic visit, where the patient is evaluated and prepared by the ERAS nurse (ideally an advanced practice nurse), a physiotherapist, a psychotherapist, an anesthesiologist and a cardiac surgeon. Minimally invasive surgical access is a central prerequisite to enable on-table extubation and early ambulation. This is supported further by standardized preoperative regional nerve block (parasternal or serratus anterior, depending on the type of access), intensive nausea prophylaxis and multimodal anesthesia and analgesia. A more detailed presentation of the institutional anesthetic and analgesic regime can be found in the Supplementary Material. Patients are extubated on-table in the operating room, provided sufficient respiratory function and stable hemodynamics, and then transferred to the IMC. The IMC provides continues invasive monitoring, non-invasive ventilation and low-dose catecholamines, but not invasive ventilation, hemofiltration or management of significant hemodynamic instability. For reasons of bed capacity between the IMC and ICU, sometimes patients are instead transferred to the ICU, where the same level of care is provided as if the patient were on the IMC. We aim to transfer patients to the general ward between 07:00 and 09:00 am on postoperative day (POD) 1, where continuous telemetry is available. Discharge home or direct admission to a cardiac rehab facility is anticipated between POD 5 and 7.

### 2.4 Statistical analysis

Results were tested for normality using the Shapiro-Wilk test. Data are presented as median and interquartile range or absolute and relative frequencies. Mann-Whitney *U*-test was used for non-parametric unpaired data. Results from statistical tests were regarded as significant when *p* < 0.05. Analysis was performed through GraphPad Prism Version 10.2.1 (GraphPad Software, Boston, MA, USA).

## 3 Results

### 3.1 Demographics

**Figure 1:**
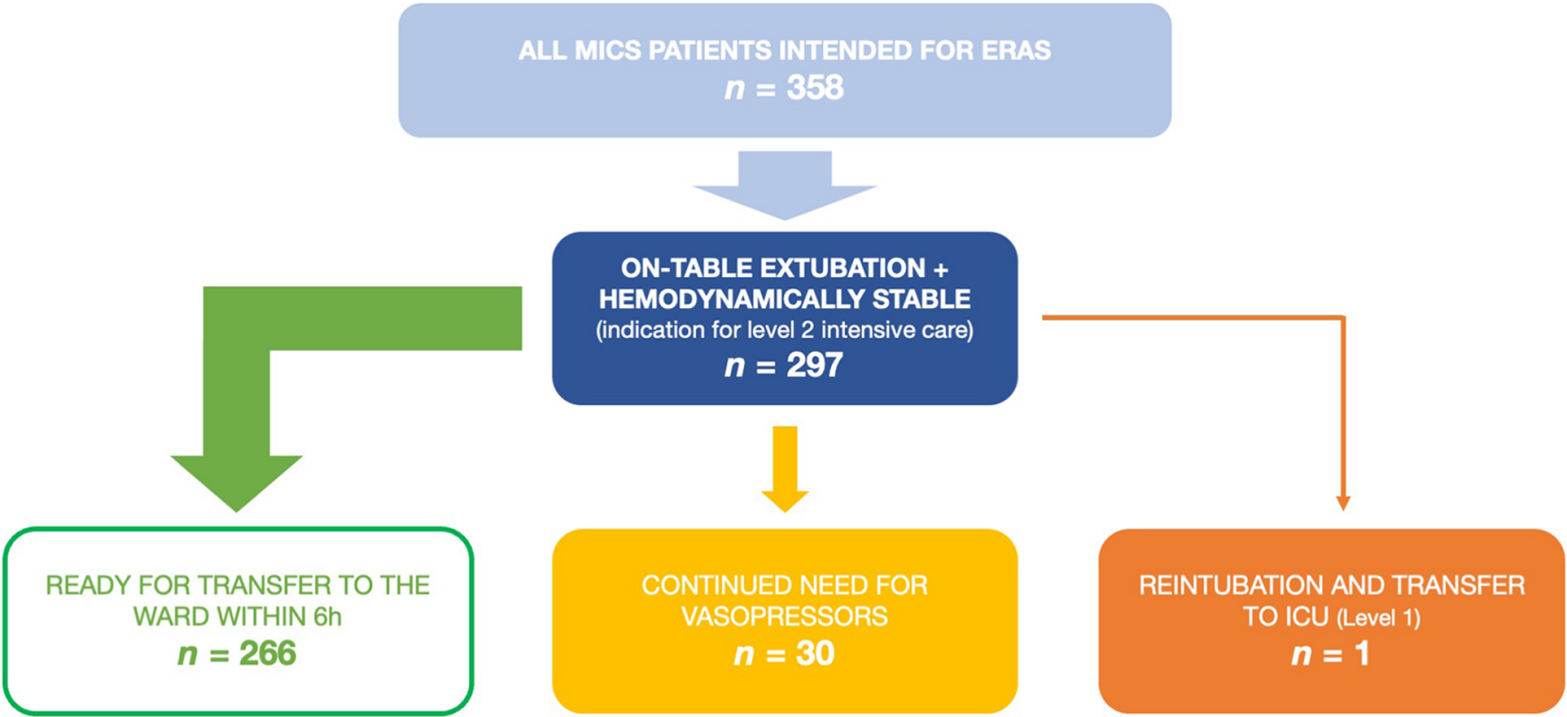
Patient flow in the presented data of patients receiving minimally invasive cardiac surgery as a part of the ERAS program. ERAS, enhanced recovery after surgery; ICU, intensive care unit; MICS, minimally invasive cardiac surgery.

358 adult patients received MICS and were deemed suitable for the ERAS protocol between 01/2021 and 03/2023 at our institution. Of these, 297 met the inclusion criteria mentioned above and therefore served as our study cohort. Patient flow within the study is illustrated in Figure 1. Patients' median age was 63 years (IQR 55–70) and 35% were female. Preoperative risk scores were fairly low, with median EuroScore II being 1.1% (IQR 0.7–1.5) and median STS Score being 0.7% (IQR 0.5–1.2). Detailed demographic data are listed in Table 1.

**Table 1:** Baseline patient characteristics.

*n* = 297	
Age (years)	63 (55–70)
Sex, male	193 (65%)
BMI (kg/m^2^)	25 (23–28)
Atrial fibrillation/flutter	78 (26%)
Coronary artery disease	57 (19%)
LVEF (%)	60 (55–60)
LVEF ≤35%	10 (3%)
Acute endocarditis	6 (2%)
Diabetes mellitus	24 (8%)
Lung disease	31 (10%)
Severe renal impairment^a^	39 (13%)
Stroke	26 (9%)
EuroSCORE II (%)	1.1 (0.7–1.5)
STS-PROM (%)	0.7 (0.5–1.2)

BMI, body mass index; LVEF, left ventricular ejection fraction; PROM, predicted risk of mortality; STS, Society of Thoracic Surgeons.

^a^ Creatinine Clearance <55 ml/min, no dialysis patients in this cohort.

All patients received MICS for valvular, aortic or atrial pathology. There was no coronary surgery in this cohort. 189 (64%) patients received anterolateral mini-thoracotomy for mitral and/or tricuspid valve surgery (*n* = 177), aortic valve replacement (*n* = 2) or other surgical procedures (*n* = 10). The remaining 108 (36%) patients had partial upper sternotomy for aortic valve repair or replacement (*n* = 79), aortic root or ascending surgery (*n* = 17) or both (*n* = 12). Surgical procedures are shown in detail in Table 2.

**Table 2:** Operative data for the patient cohort.

Access	Operation	
Right lateral minithoracotomy (*n* = 189)	Mitral valve repair/replacement	167 (88%)
	ASD repair	5 (3%)
	Myxoma resection	5 (3%)
	Tricuspid repair/replacement	2 (1%)
	Combined MV/TV surgery	2 (1%)
	Combined MV and ASD repair	6 (3%)
	Aortic valve replacement	2 (1%)
Partial upper sternotomy (*n* = 108)	Aortic valve replacement	64 (59%)
	Aortic valve repair	15 (14%)
	David/Bentall	12 (11%)
	Ascending aortic aneurysm repair	5 (5%)
	Aortic valve and ascending aorta	12 (11%)

ASD, atrial septal defect; MV, mitral valve; TV, tricuspid valve.

Patients were transferred to the general ward from the IMC/ICU after a median of 22 h (IQR 20–24).

### 3.2 Occurrence and timing of major complications

Major complications were very rare in this cohort. In total, two (0.7%) MACE events occurred: one myocardial infarction and one stroke. One was a partial occlusion of the circumflex artery after mitral valve repair, which became apparent through transient ST-elevations on telemetry on POD 2. The patient was referred to coronary angioplasty and received a drug-eluting stent. The single stroke was likewise diagnosed on POD 2 when the patient complained of persistent vertigo and ataxia during physiotherapy and was taken for cranial computed tomography scan after neurological evaluation.

There was a total of four (1.3%) postoperative bleeding events requiring reexploration (see Table 3). Of these, only a single instance of bleeding occurred while the patient was still on the IMC but beyond the 6 h mark, all others took place >24 h postoperatively after transfer to the general surgical ward. Two out of four bleeding events were subacute, so that surgical intervention was required to evacuate the hematoma for infection prophylaxis.

**Table 3:** MACE, bleeding events and potentially fast-track-associated complications (reintubation, readmission to ICU) and their timing.

	Clinical event	Time to event	After transfer to ward from IMC
Patient 1	STEMI due to iatrogenic RCx-occlusion	POD 2	Yes
Patient 2	PICA stroke, subacute hemothorax	POD 2–3	Yes
Patient 3	Subacute hemothorax	POD 5	Yes
Patient 4	Acute hemothorax, hemorrhagic shock	20 h	No*
Patient 5	Acute hemothorax	25 h	Yes
Patient 6	Progressive respiratory insufficiency requiring reintubation due to hypercapnia	2 h	No
Patient 7	Unclear hypotension	POD 2	Yes
Patient 8	Respiratory insufficiency requiring NIV due to hypervolemia	POD 2	Yes

Patient #4 was the only event which took place >6 h postoperatively but before transfer to the ward.

STEMI, ST-elevation myocardial infarction; POD, postoperative day; IMC, intermediate care unit; RCx, Ramus circumflex; PICA, posterior inferior cerebellar artery.

### 3.3 Occurrence and timing of fast-track-associated complications

Aside from the detection of major complications, postoperative critical care also addresses usually minor, transient sequelae to otherwise uncomplicated surgery such as mild vasoplegia, mild respiratory insufficiency and pain. Within the patient cohort, after initially successful on-table extubation, there was one instance of reintubation due to progressive hypercapnia approximately 2 h postoperatively (patient #6, see Table 3).

At our institution, the first-line vasopressor is norepinephrine and the first-line inotrope is milrinone. 259 (87%) patients required no hemodynamic support at the end of surgery and remained that way until transfer to the ward. All following patients received either only norepinephrine or norepinephrine and milrinone, no other catecholamines were used: 7 patients (2%) required low-dose vasopressors initially but could be weaned within 6 h and were persistently normotensive until transfer to the ward on POD 1. Another 6 (2%) patients required more than 6 h to be weaned off vasopressor support, but remained persistently normotensive after 12 h postoperatively. The remaining 25 (9%) patients either needed vasopressors within the first 12 h despite being initially normotensive immediately after surgery or had persistent vasopressor need from the time of the surgery. The last two categories, 31 patients (10%) in total, would therefore not have been candidates for transfer to the ward six hours postoperatively. In total, 266 (90%) patients would have been normotensive without need for vasopressors at the six hour mark. This is illustrated in Figure 1.

The 31 patients requiring extended vasopressor support were significantly older (median 70 vs. 62 years; IQR 63–76 vs. 54–70; *p* < 0.001), had higher STS PROM Score (median 1.1 vs. 0.7%; IQR 0.7–2.0 vs. 0.5–1.1; *p* < 0.001) and had significantly lower left ventricular ejection fraction (median 60 vs. 60%; IQR 40–60 vs. 56–60; *p* = 0.04), as well as lower preoperative creatinine clearance (median 66 vs. 89 ml/min; IQR 56–90 vs. 70–116; *p* < 0.001) compared to the 266 patients who did not.

The aforementioned patient #6 (see Table 3), who had to be reintubated for hypercapnia, also required vasopressors from the time of surgery and remained in need of hemodynamic support for more than 12 h. Patient #4 (see Table 3) only developed clinical and paraclinical signs of hemorrhage, including need for vasopressors, between the 6th and 12th postoperative hours.

**Figure 2:**
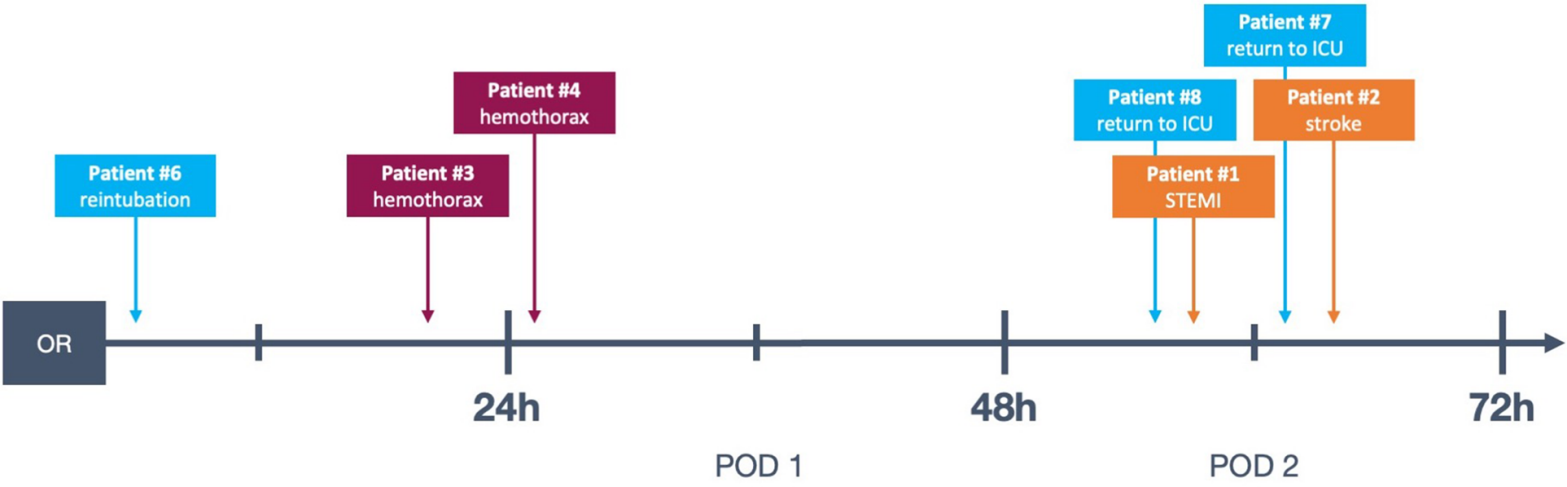
Timeline of complications in the presented data, see also Table 3. Patient #5 not represented because the complication took place on POD 5. OR, operating room; POD, postoperative day; STEMI, ST-elevation myocardial infarction; ICU, intensive care unit.

There were two instances of return to ICU after the patients had been transferred to the ward, one for respiratory insufficiency requiring non-invasive ventilation and one for transient hypotension. Both of these events took place on POD 2 (see Table 3 and Figure 2).

In conclusion, of the 297 patients in our cohort, 266 (90%) would have been normotensive without medical support, had no respiratory issues 6 h after surgery and, therefore, would have been suitable for transfer to the general ward.

## 4 Discussion

### 4.1 Patient safety in fast track

Based on our experience, if patients can be successfully extubated on-table and are hemodynamically stable at the end of surgery, major postoperative complications are very rare. Given that the majority of postoperative complications occurred within six hours or significantly later than 16 h postoperatively and after transfer to the ward, there appears to be potential to safely reduce intensive monitoring to a 6 h period. The data published by Haanshoten et al. reflects this: In their Fast Track protocol, patients remained on the PACU for an average of 7 h postoperatively, before transfer to the ward on the day of surgery. This did not impact patient safety (13). More generally, the results of 26 studies concerning Fast Track protocols in cardiac surgery for early extubation, as well as measures to reduce ICU and hospital lengths of stay were analyzed by a Cochrane review in 2016 and deemed safe for low to moderate risk patients (15). Nevertheless, given the relevant risk of severe postoperative morbidity in the field of cardiac surgery, a postoperative observation period to allow for detection of potential major complications appears to be necessary. There does not, however, seem to be a need for this postoperative monitoring period to take place on a level 1 (high care) ICU - alternatively, monitoring in a PACU or similarly equipped setting was described in different studies as just as feasible without compromising safety (8, 13, 16). Our results concur with this, since the majority of our patients met the criteria for being transferred to IMC (level 2) rather than a traditional cardiac surgical ICU. Additionally, insofar as further de-escalation of not only the setting but the duration of monitoring is concerned, discharge from ICU, IMC or PACU on the day of surgery has been found to be safe even after non-MICS procedures such as coronary artery bypass grafting and aortic valve replacement via full sternotomy (8, 10, 13). In summary, our results are in line with the aforementioned conclusions of prior studies. There appears to be a potential to not only decrease the length of intensive monitoring after cardiac surgery, but also change where this monitoring takes place—from standard highest care ICU monitoring towards lower care units like PACU—without negatively impacting patient safety.

Additionally, in an era characterized by diminishing financial and personnel resources in healthcare, alongside escalating unpredictable burdens like pandemics, resource and cost saving are crucial tasks in contemporary patient care. There is potential for considerable resource and cost reduction in deescalating and shortening postoperative critical care within the realm of cardiac surgery. These savings can subsequently benefit other patients, thereby addressing critical needs amidst resource constraints and fostering more efficient allocation of healthcare resources.

### 4.2 Bleeding

All four episodes of bleeding requiring reexploration took place in patients who had received anterolateral mini-thoracotomy and experienced chest wall bleeding. Notably, in our study cohort, no acute postoperative bleeding leading to cardiac tamponade and subsequent emergency surgical revision occurred. Of the registered bleeding events, only one took place while still on the IMC, when the patient (Patient #4, see Table 3 and Figure 2) was taken for thoracic computed tomography after suspicion for hemothorax was raised via ultrasound. This took place approximately 20 h postoperatively, and is therefore a relevant complication discovered during an extended monitoring period on the IMC. This was, however, the only instance of bleeding which was diagnosed while a patient was still on the IMC: The three other bleeding events presented in a more subacute manner and were diagnosed on the general ward (see Table 3 and Figure 2, patients #2, #3 and #5).

It is possible that access-related bleeding represents a pitfall of Fast Track after procedures via mini-thoracotomy. It appears viable, however, to mandate ultrasound of the pleural and pericardial spaces as well as chest x-ray before transfer out of a PACU, in order to recognize possibly latent effusions before transfer to the ward. If no significant bleeding from the chest drain is noted, and ultrasound as well as x-ray exams are unremarkable at the 6 h mark, it appears very unlikely that significant bleeding would go unrecognized before transfer to the ward. Furthermore, after transfer to the ward, patients continue to be monitored, with particular emphasis on chest drain output volume and quality, vital signs, and regular blood gas analysis. This ongoing monitoring aims to detect any delayed complications that may arise, ensuring comprehensive postoperative care beyond the immediate recovery period.

### 4.3 On-table extubation

A distinctive feature of our planned Fast-Track program, and a fundamental element of the already established ERAS protocol, is on-table extubation. The utilization of anterolateral mini-thoracotomy and mini-sternotomy in MICS preserves the integrity of the chest wall, thereby facilitating improved respiratory function and enabling routine on-table extubation. The importance of using minimally-invasive surgical access routes when implementing early or on-table extubation is underlined by previous studies, which have demonstrated that MICS in general is associated with shorter ventilation times, shorter ICU stays and decreased hospitalization when compared to median sternotomy (17–19). Alongside the positive impact of MICS, Fast-Track protocols can further reduce ventilation time and ICU length of stay (8, 10). Smaller studies showed on-table extubation to be safe in cardiac surgery even after median sternotomy as early as the late 1990s and early 2000s (20–23), yet Fast-Track protocols for cardiac surgery often target early extubation (usually within 2–4 h) instead of immediate extubation (8, 10, 16). On-table extubation, likewise, is associated with shorter ICU and hospital lengths of stay (24), even when compared to early extubation protocols (25). This illustrates the additional benefit of not only early, but immediate postsurgical cessation of anesthesia and invasive ventilation can have for patient recovery. Thus, by incorporating routine on-table extubation into our Fast Track approach, we can further minimize anesthetic duration and potentially eliminate the need for postoperative ICU admission altogether.

### 4.4 A fast-track pathway for MICS

The aim of this study is to show the feasibility of a Fast Track model in the setting of our ERAS program for MICS patients. Our data show that in the setting of our institutional ERAS program, after successful MICS with no significant intraoperative complications, 90% of patients would have been stable for transfer to the ward 6 h postoperatively.

Ender et al. (8) published the results of their Fast Track program in 2005, which demonstrated a safe model for a direct-admit PACU, where patients would be rapidly extubated and then remained on the PACU for a median of 4 h until transfer to IMC, which provided continuous telemetry but not invasive ventilation or vasopressors. At the same institution, a small randomized controlled trial showed that transfer to PACU reduced ventilation time and the duration of intensive monitoring without compromising patient safety (26). A study investigating 5,367 patients after coronary surgery or aortic valve replacement published by Haanshoten et al. (13), also implementing a Fast Track pathway through a direct-admit PACU, showed a 7 h average length of stay there. 84% of those patients could be successfully transferred to the ward immediately afterwards, where continuous telemetry, non-invasive monitoring of oxygen saturation and blood pressure was provided until the following day. There was likewise no negative impact on patient safety. This is particularly noteworthy considering that these data involve patients who received procedures via median sternotomy, which is significantly more invasive compared to MICS procedures, and consequently complicates early extubation due to a greater impact on the stability of the thorax.

These favorable results from prior research as well as our own data demonstrate that 6 h of intensive monitoring in a PACU might be feasible, provided careful patient selection and institutional planning. Patients who would be included in the Fast-Track scheme at our institution would be those who met the eligibility criteria for our ERAS program. Additional exclusion criteria would then have to be applied, for example exempting those with advanced heart failure or chronic kidney disease, as these are factors which have been shown to be associated with failure of Fast-Track protocols (7, 12, 13, 27). Select patients would undergo MICS, followed by on-table extubation before transfer out of the operating room. Provided the intraoperative course had been uncomplicated, subsequently, they would be transferred to the PACU for 6 h of rigorous monitoring. If patients met the institutional requirements for transfer to the ward at 6 h postoperatively, they would be moved there accordingly. These requirements would be for the patient to be alert and oriented, normotensive without vasopressors or inotropes, with acceptable gas exchange on no more than 5 L/min of oxygen, sufficient pain control, chest drain output of <50 ml/h and unremarkable ultrasound of the pericardial and pleural spaces. Non-invasive monitoring, including continuous telemetry, continuous pulse oximetry, intermittent non-invasive blood pressure measurements and monitoring of diuresis and chest drain output, would be continued until the morning of POD 1. Continuous wearable telemetry would then be maintained until the removal of pacemaker wires, typically around POD 3, or as deemed clinically appropriate.

A point of concern in our data are the very small proportion of patients (*n* = 5; 2%), who developed new vasopressor need beyond 6 h postoperatively. All five of these patients required very low doses of vasopressors at 12 h postoperatively due to mild but progressive hypotension. There were no instances of acute hemodynamic collapse amongst these patients. These patients would, however, be identified by the monitoring protocol on the general ward and could then be transferred to IMC or ICU if required. This would involve sufficient availability of ICU bed capacity as well as close coordination with ICU staff in order to escalate care for Fast Track patients at any time, should it become necessary. If there was concern for any vasopressor use postoperatively, and if patients with any degree of vasopressor need within the first 6 h were excluded from Fast Track transfer to the general ward, 87% of the cohort would have been persistently normotensive without any hemodynamic support at any time, and therefore eligible for transfer.

### 4.5 Limitations

As a retrospective observational single-center study, our results should be interpreted with caution. The findings were influenced by the specific protocols established by our hospital's multidisciplinary team, consisting of anesthesiologists and cardiac surgeons. Furthermore, the data presented remains in preparation of the imminent establishment of a PACU pathway at our institution and therefore does not yet reflect real-world experience but only the investigation of a theoretical concept. Following its implementation, the Fast Track pathway would have to be closely evaluated in a clinical setting. Furthermore, patient eligibility and selection would have to be highly rigorous in order to maintain safety standards.

## 5 Conclusions

In our selected ERAS patient cohort, major postoperative complications were rare when patients were successfully extubated on-table and hemodynamically stable at the conclusion of surgery. On-table extubation, a fundamental component of our planned Fast-Track program, has been shown to be safe. Considering the results of this study, there might be potential to reduce and deescalate postoperative intensive monitoring to a 6-hour period in a PACU, given that most complications occur within six hours postoperatively or considerably later, usually after transfer to the general postsurgical ward. This might make it possible to completely avoid postoperative ICU/IMC admission in well selected patients. While the results from this retrospective analysis are encouraging, this treatment pathway is still largely theoretical and requires further study before safety and efficacy can be established.

## Data Availability

The raw data supporting the conclusions of this article will be made available by the authors, without undue reservation.
