# Supplementary material for: Feasibility of deescalating postoperative care in enhanced recovery after cardiac surgery
Source: Front Cardiovasc Med. 2024 Aug 12;11:1412869. doi: 10.3389/fcvm.2024.1412869 (PMC11345171; doi:10.3389/fcvm.2024.1412869)
Supplement: Supplementary file 1 [file Datasheet1.pdf]

Supplementary material for
"Feasibility of Deescalating Postoperative Care in Enhanced Recovery After Surgery"

Sina Stock[1]†*, Sarah Berger Veith[1]†, Theresa Holst[1], Sahab Erfani[1], Julia Pochert[2], Christian Dumps[2], Evaldas Girdauskas[1]

† These authors contributed equally to this work and share first authorship
[1]Department of Cardiac and Thoracic Surgery, University Hospital Augsburg, Augsburg, Germany
[2]Department of Anesthesiology and Intensive Care Medicine, University Hospital Augsburg, Augsburg, Germany

*** Correspondence:**
Sina Stock
sina.stock@uk-augsburg.de

Perioperative anesthetic and analgesic regimen for ERAS in cardiac surgery

Perioperative anesthetic regimen:
For the induction of general anesthesia, sufentanil, thiopental and rocuronium are used. After endotracheal intubation but before surgery, either parasternal block (for mini-sternotomy) or serratus anterior block (for mini-thoracotomy) with 0.2% ropivacaine is performed in order to reduce intra- as well as postoperative analgesic requirements. Intraoperatively, amesthesia is maintained using remifentanyl, clonidine and sevoflurane. Clonidine (30 – 90 µg/h) is continued postoperatively to help ease postoperative agitation when necessary. Upon extubation, analgesia is provided with piritramide.
For PONV prophylaxis, all patients receive dexamethasone after induction of anesthesia as well as granisetrone and dimenhydrinate upon extubation.
Extubation is performed when the patient is normothermic, normovolemic, has adequate gas exchange and respiratory drive, sufficient protective reflexes and is hemodynamically stable on no more than moderate doses of catecholamines. We have found neither postoperative agitation or drowsiness to be prohibitive for on-table extubation after cardiac surgery

Standard multimodal analgesic regimen:
Patients receive metamizole and piritramide PRN while on IMC or ICU on the day of surgery.
Upon POD 1 patients receive standard 1g metamizole every 8h and 10mg extended-release oxycodone/naloxone twice daily, as well as pregabalin between 25 and 100mg twice daily if they received thoracotomy. On-demand analgesia is usually achieved by hydromorphone. Opiates are generally discontinued between POD 3 and 5 and always prior to discharge from the hospital. If adequate analgesia cannot be achieved through oral medication, repeat regional nerve blocks can be administered, although instances of severe postoperative pain have been rare in our experience.
Chest drains are removed on the day of surgery if output is < 500 ml in the first 8 hours and no air leak is noted. Otherwise chest drains are removed on the morning of POD 1. Early or even very early removal of chest drains appears to reduce need for analgesia, allowing patients to be opiate-free earlier.
